# Hypoxia Pathways and Cellular Stress Activate Pancreatic Stellate Cells: Development of an Organotypic Culture Model of Thick Slices of Normal Human Pancreas

**DOI:** 10.1371/journal.pone.0076229

**Published:** 2013-09-30

**Authors:** Vinciane Rebours, Miguel Albuquerque, Alain Sauvanet, Philippe Ruszniewski, Philippe Lévy, Valérie Paradis, Pierre Bedossa, Anne Couvelard

**Affiliations:** 1 Pancreatology Department, Beaujon Hospital, AP-HP, Paris-Diderot University, Clichy, France; 2 Inserm U773-CRB3, Paris-Diderot University, Paris, France; 3 Pathology Department, Beaujon Hospital, AP-HP, Paris-Diderot University, Clichy, France; 4 Pancreatic Surgery Department, Beaujon Hospital, AP-HP, Paris-Diderot University, Clichy, France; 5 Pathology Department, Bichat Hospital, AP-HP, Paris-Diderot University, Paris, France; University of Missouri, United States of America

## Abstract

Pancreatic stellate cells (PSC) are involved in fibrogenesis and oncogenesis by modulating the extracellular matrix.

**Aim:**

To evaluate the effect of cellular stress on PSC activation using a model of normal human pancreatic tissue slices culture preserving the microenvironment.

**Methods:**

Thin sections (300μm) of normal human pancreas were cultured under hyperoxia (90% O2) during 72 hours. Viability and morphological analysis were performed at baseline, H24, H48 and H72. Cell differentiation (insulin, trypsin, CA9 and CK7), hypoxia (HIF1-α), apoptosis (caspase-3), proliferation (Ki67), TGF-β expression and PSC activation (smooth muscle actin (SMA), nestin) were assessed using immunostaining, longitudinally. Control experiments were performed under normoxic conditions (21% O2).

**Results:**

Thirty sections per specimen (n=10) were cultured. Hypoxia pathways were activated by the higher expression of HIF1-α at H48 and H72. Apoptosis was limited with only rare acinar cells expressing of the caspase-3 at 48 and H72 (NS). Morphological analysis showed gradual appearance of acinoductal metaplasia, proven by CK7 expression and ductal phenotype of dedifferentiated acini. Transdifferentiation of PSC was shown by de novo SMA immunochemistry at H24 and H48. Expression of Ki67 index identified significant proliferation of activated PSC (double immunostaining Ki67-SMA) at H48 and H72 (p=0.02). In vitro culture of normal human pancreas thin sections is feasible with optimized cell viability at 72 hours. This model of culture in hyperoxic conditions provides evidences that cellular stress may rapidly induce transactivation of PSC with ducto-acinar metaplasia.

## Introduction

The role of the pancreatic stellate cells (PSC) in the pancreatic fibrogenesis process is now well identified. By analogy to hepatic stellate cells, vitamin A storing cells were first described in the pancreas, by Watari et al in 1982 [1]. In 1998, the star-shaped cells in the pancreas were identified and called PSCs [2,3]. Characteristics and distinguishing markers of PSC include storage of retinyl palmitate, expression of cytoskeletal marker proteins (vimentin, desmin and glial fibrillary acidic protein (GFAP)) and a phenotypic transition to an extracellular matrix producing myofibroblast-like cells (α-smooth muscle actin, SMA) [4]. In case of pancreatic injury, the activation of PSC results from profibrogenic mediators such as inflammatory cytokines and oxidant stress. This activation greatly increases synthesis of extracellular matrix proteins such as collagens and structural glycoproteins [4]. Over the past few years, several studies have reported the main role of PSC in the fibrogenesis process in acute and chronic pancreatitis due to the imbalance between fibrogenesis and matrix degradation. Moreover, recent studies suggested that PSC play an important role in the pathophysiology of pancreatic cancer because of specific interactions: activated PSC were identified in fibrotic areas of pancreatic cancer and seem to influence cancer growth and proliferation. Pancreatic cancer cells produce mitogenic and fibrogenic factors (such as TGF-1, PDGF), which may promote the activated PSC phenotype. In a positive feedback, activated PSC release stimuli that mediate tumor growth, invasion and metastasis[5-8].

Hypoxia phenomena are believed to play a major role in PSC activation, which is inversely correlated with vascular density. Pancreatic adenocarcinoma as well as chronic pancreatitis are characterised by hypoxia phenomena and fibrosis[9,10]. When human PSCs were cultivated in vitro under hypoxia, activity of PSCs increased and the secretion of periostin, type I collagen, fibronectin, and vascular endothelial growth factor (VEGF) was doubled. Under hypoxia, PSC contributed to the fibrotic/hypoxic milieu through abnormal extracellular matrix deposition[9,11]. However, these results were obtained in *in vitro* models of PSC culture. These models do not take into account the pancreatic microenvironment. Furthermore, the very early step in PSCs activation under cellular stress in normal pancreatic tissue remains still unknown.

Indeed, models for studying PSC are mainly cultures of murine or human PSC lines or immortalised PSC. Differences between species and donors could make it difficult to interpret the results; moreover these models do not closely approximate the *in vivo* situation in patients[5,12]. Models of thin tissue slices (precision-cut slices) culture were developed to analyse metabolism and physiopathological mechanisms in normal organ tissue such as the liver and the kidney[13-15]. Tissue slices contain all cell types of the tissue in their natural environment, with intercellular and cell-matrix interactions remaining intact to more closely approximate the *in vivo* situation.

The aims of this study were to develop a model of human pancreatic organotypic culture, in order to analyze morphological changes and to evaluate the early activation and proliferation of PSC in their normal microenvironment under cellular stress conditions.

## Materials and Methods

### Patients and tissue sample selection

Human pancreatic samples were collected from surgical specimens of patients operated on in 2011 and 2013 in Beaujon Hospital, Clichy, France. All patients underwent surgery for benign pancreatic lesions such as neuroendocrine tumors (n=10), mucinous cystadenoma (n=4) and solid pseudopapillary tumor (n=1). A total of 15 tissue samples were selected. A specific written information was given and explained to all patients. Written informed consent was obtained for all patients before surgical resection and archived in the medical file. An institutional review board (CEERB, comité d’éthique en recherche biomédicale du Groupe hospitalier universitaire Nord) approval for the study design and the ethical measures was obtained on May 19, 2011 (IRB00006477- Study reference: 11-058). The ethical committee reviewed all the written consents.

### Preparation of precision-cut human pancreatic slices

Pancreatic specimens were retrieved from the surgical wing as freshly as possible after resections to prevent warm ischemia. Specimens were carried in the pathological unit on ice (at 4°C). Cylindrical core samples were taken 2 cm apart from the benign tumor by a punch (8 mm diameter). All steps up to incubation were performed on ice (at 4 °C) and the core samples were submerged in an iced buffer during the procedure of slices preparation.

A Krumdieck Tissue Slicer (TSE Systems, Inc, Midland, USA) was used to perform fresh pancreatic tissue slices under aseptic conditions. The microtome, the reservoir and all instruments were sterilized before use to prevent microbiotic contamination. The manipulations were realized under flow hood. Three hundreds µm thick slices were prepared at a rate of one slice every 5 seconds by continuous oscillations of the blade. The slicer operated submerged in a specific ice-cold and oxygenated slicing buffer (Hank's Balanced Salt Solution, HBSS, Invitrogen) supplemented with glucose (5 mM) and antibiotics (Penicillin 25 UI/ml)). A minimal number of 30 slices was performed for each specimen. After slicing the cores, the slices were removed from the glass trap, collected and placed immediately on ice. The selection of the slices was realized on the basis of appearance, to obtain equal thickness and uniform colour. The first two sections were immediately stained with haematoxylin and eosin (H&E) and were all analyzed by a pathologist (AC). The goal was to select pancreatic samples (quantity and quality of pancreatic tissue) and to confirm the absence of relevant lesions and the normality of the tissue at microscopic examination.

### Conditions of incubation

Slices were loaded onto specific titanium screen holders, which were transferred to standard tissue culture six-well plates. Plates were placed on a rocker platform to alternately expose the slices to the atmosphere of the tissue culture incubator or dip them in the culture medium in order to provide optimal oxygenation and nutrient delivery to tissue. Plates with culture medium were prewarmed and oxygenated by placing them in the incubator (Binder, Germany) at 37 °C for at least 30 min. Slices were gently agitated in the humidified incubator at 37°C, under hyperoxic conditions with 5% CO2 and 90% O2 (n=10 specimens). A control analysis was performed and slices from 5 specimens were cultured under normoxic conditions. Slices were placed and agitated in the humidified incubator at 37°C, with 21% O2. In the two conditions of culture, DMEM/F-12 (+ l-glutamine) was used as medium supplemented by insulin (0,35 μM), dexamethasone  (0,1μM), fetal calf serum (5%), antibiotics (Gentamicin sulfate (50 mg ml −1; Invitrogen) and Penicillin (25 UI/ml)), HEPES, heptanoic acid (2 mM) and glutathione (1 mM). Medium was refreshed at 1 hour and every 24 hours. Slices were cultured for 72 hours. Analysis of sections after H&E and immunohistochemical (IHC) staining was performed at baseline, H24, H48 and H72.

### Viability test

Viability of tissue was analyzed by a MTT (3-(4,5-diméthylthiazol-2-yl)-2,5 diphenyltetrazolium bromide) test at each step of the procedures at baseline, H24, H48 and H72. The culture medium was removed at the different incubation times and the pancreatic slices were incubated with MTT (2 mg/ml) for 1h at 37°C. After 1h incubation, the MTT solution was removed, each pancreatic slice was rinsed twice with cold PBS (500 µl), and the formazan product was extracted and diluted with DMSO (500 µl) by gentle agitation during 20 minutes. The absorbance was measured at a wavelength of 570 nm (Spectramax M5).

### Pathological examination

After culture at each step (baseline, H24, H48 and H72), pancreatic slices were routinely formalin-fixed and paraffin-embedded. Sections (4μm thickness) were stained with haematoxylin–eosin (H&E) for pathological examination, by light microscopy by two investigators (AC and VR) for each case. PSC were defined as interacini cells with a central cell body and long cytoplasmic projections extending along the base of adjacent acinar cells. PSC activation was analyzed by α-smooth muscle actin (α-SMA), GFAP and Nestin immunostaining and PSC proliferation was evaluated by Ki67/α-SMA antibodies double immunostaining. Acinoductal metaplasia was defined as the replacement of acinar cells by duct cells, or a switch from differentiated acinar cells to differentiated/functional ductal cells. The location and the distribution of the dedifferentiated cells were specifically noticed. CK7 immunostaining was performed to evaluate and confirm the ductal phenotype and the maturation stages of the dedifferentiated acinar cells. Cell viability was studied by the evaluation of apoptosis (activated caspase-3 expression), by the estimation of the neuro endocrine (islets of Langerhans) and exocrine (acinar) cell activity and differentiation (insulin and trypsin staining) and by the evaluation of hypoxia pathways activation (Carbonic Anhydrase 9 (CA9) and HIF1-α immunostaining). The TGF-β (Tumour growth factor) and the collagen I expressions were studied at each step.

### Immunohistochemical Analysis

#### Techniques

Four µm sections were obtained from pancreatic slices. Immunohistochemistry was performed with an automated immunohistochemical stainer according to the manufacturer’s guidelines (Streptavidine-peroxidase with an automate Ventana, Benchmark, USA). A double staining was performed using Ultraview universal alkaline phosphatase red detection and Ultraview DAB kits (Ventana, Benchmark, USA) to confirm antigen co localization in some cases. Immunostaining was performed after the slides were dewaxed and rehydrated. Antigen retrieval was conducted by pretreatment at a high temperature. PBS was substituted for the primary antibody and used as a negative control. The slides were immunolabeled with monoclonal antibodies against Ki-67 (1:200, clone MIB-1, Dako, USA), α-smooth muscle actin (1:500, α-SMA, clone 1A4, Dako, USA), GFAP (1:2000, clone 6F-2, Dako, USA), Nestin (1:500, Santa Cruz), CA9 (1:200, Novus Biological, Littleton, USA), HIF1-α (1:250, clone L5-B674, Lifespan, Biosciences, USA), CK7 (1:500, clone 50V-TL, Dako, USA), insulin (1:25, Dako, USA), trypsin (1:300, Immunotech, USA), CD31 (1:200, clone JC/70A, Dako, USA), CD34 (1:500, clone QBEND 10, Immunotech, USA), caspase-3 (1:200, clone C92-605, BD Biosciences, USA), TGF-β (1:40, clone TGFB17, Abcam, Cambridge, UK) and collagen I (1:50, clone NB600-408, Novus Biologicals, Littleton, USA).

#### Evaluation of the staining, scoring methods

All sections were evaluated by two investigators (AC and VR). The estimation of immunoreactive cells was performed in the entire area of the evaluated slice. The tissue viability was assessed by apoptosis evaluation by counting the total number of caspase-3 positive acinar cells per high-power field (HPF x 40 objective) in a minimum of 3 HPFs (3HFP=0.75 mm^2^). The result corresponded to the mean number of positive acinar cells per field (i.e per 0.25mm^2^). Pancreatic tissue differentiation was evaluated in a semiquantitative fashion by evaluation of the expression intensity of insulin in islet cells, trypsin in acinar cells and CK7 in ductal and dedifferentiated acinar cells. The same score estimated the TGF-β and CA9 expression. The intensity of staining was evaluated as negative scored as 0, weak scored as 1, moderate scored at 2 and strong scored as 3. The activation of the hypoxia pathways was assessed by the expression of the HIF1-α and the CA9 in acinar cells according to the same semiquantitative score. PSC activation was evaluated according to the number of positive PSC staining for α-smooth muscle actin, nestin and GFAP. The score corresponded to 0: no positive PSC staining per 3 HPF (x40 objective); 1: rare positive PSC per 3 HPF and 2: several positive PSC per 3 HPF. PSC proliferation was assessed by Ki67 positive staining, as the mean of the total number of Ki67 PSC per HPF (HPF x 40 objective) counted in a minimum of 3 HPFs. PSC proliferation was confirmed by a double Ki67/SMA immunostaining, showing nuclear red staining for Ki67 and cytoplasmic brown staining for SMA in the same cells (Roche-Ventana, USA). The collagen I expression was analysed by immunochemistry. For each slide, the proportion of positive area was evaluated by the CaloPix software (Tribvn, USA) in three randomized area. The ratio of the proportion of positive area versus the total selected area was performed. The pattern of expression (cytoplasmic, membranous, or nuclear) was noted for each antibody.

### Statistical analysis

General characteristics were expressed as medians and ranges or percentages. Comparison of general characteristics, pathological and immunohistochemical data were performed between the groups using the Kruskall-Wallis test for continuous data and the Chi^2^ test or Fisher’s exact test for categorical data when necessary. Data were analyzed with SAS 9.1 statistical software for Windows (SAS Institute Inc., Cary, NC, USA). All statistical tests were two-sided; p <0.05 was considered to be statistically significant.

## Results

### Microscopic examination

**Figure 1:**
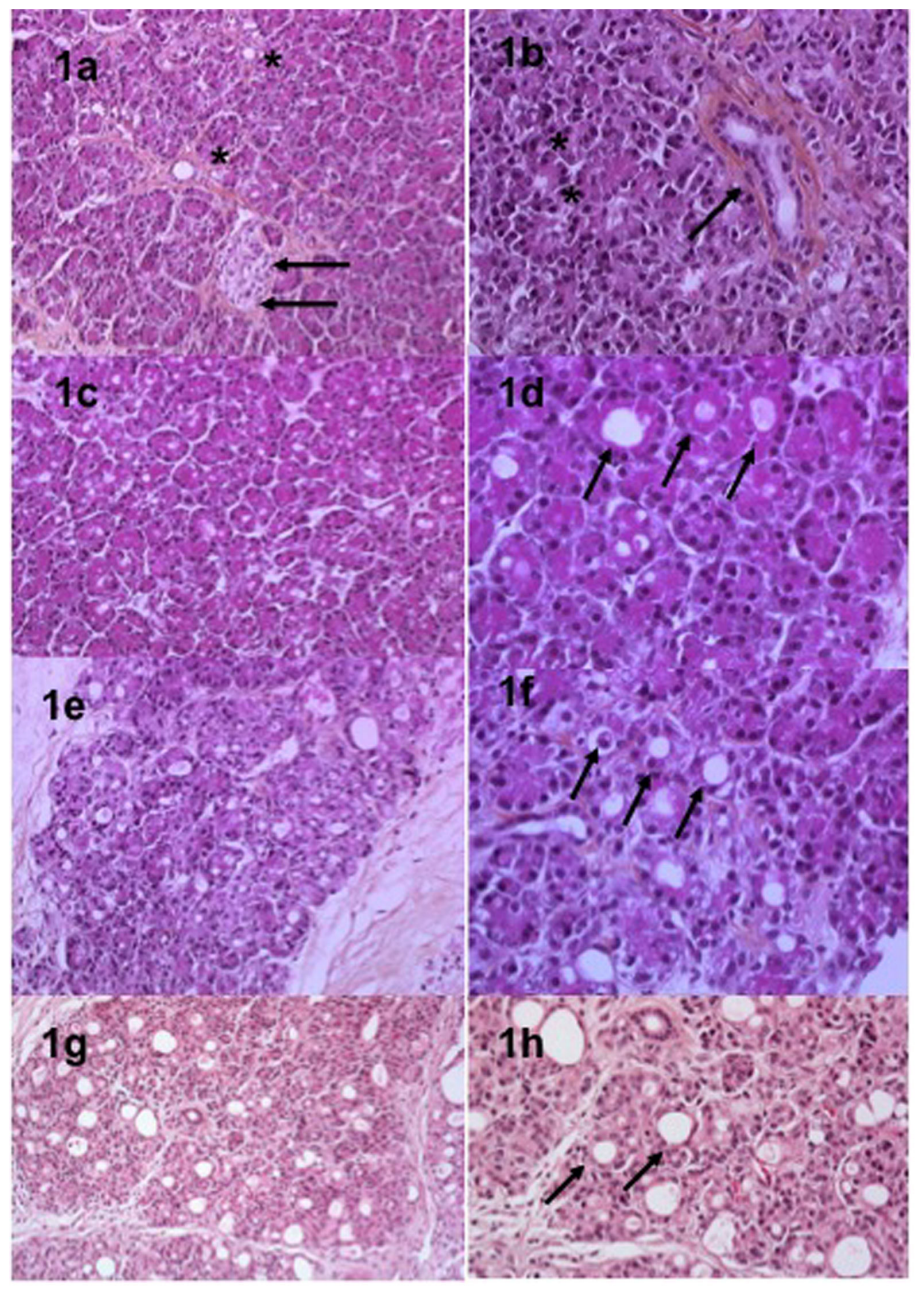
Microscopic examination of tissue slices at baseline (1a, 1b), H24 (1c, 1d), H48 (1e, 1f) and H72 (1g, 1h) in hyperoxic conditions. Different types of cells were observed: pancreatic ducts (black asterisks, 1a), islets (black arrows, 1a), acinar cells (black asterisks, 1b) and endothelial cells (black arrows, 1b). Acinoductal metaplasia (black arrows, 1d, 1f and 1h) was observed with a gradual appearance during the culture process.

The normal pancreatic architecture was preserved at each step (Figure 1). The different cell types are identified in the Figure 1. Under 90% O2 condition of culture, foci of necrosis were observed in all cases at H72 and represented less than 10% of the slice. A gradual acinoductal metaplasia was observed in 8/10 cases with an increasing incidence along the culture process. This phenomenon was mainly observed at the periphery of the slices at H24 and progressively increased in the central areas of the slices at H48 and H72 involving up to 50% of the total slices area in 6/8 cases (Figure 1). Under normoxic conditions, foci of necrosis were less prevalent at H72 and represented less than 5% of the area of the slices. The acinoductal metaplasia was observed in 5/5 of the cases. The appearance was gradual without area of predisposition (peripheral or central area). At H72, this metaplasia involved less than 50% of the total area of slices.

### Tissue viability

Tissue viability was first assessed by pathological exam at each step of the culture for all cases. Parameters of viability included cell swelling, necrosis and nuclear pyknosis. All types of cells including acinar and ductal cells, islets of Langerhans, endothelial and nerve cells were clearly distinguished at each step (Figure 1).

**Figure 2:**
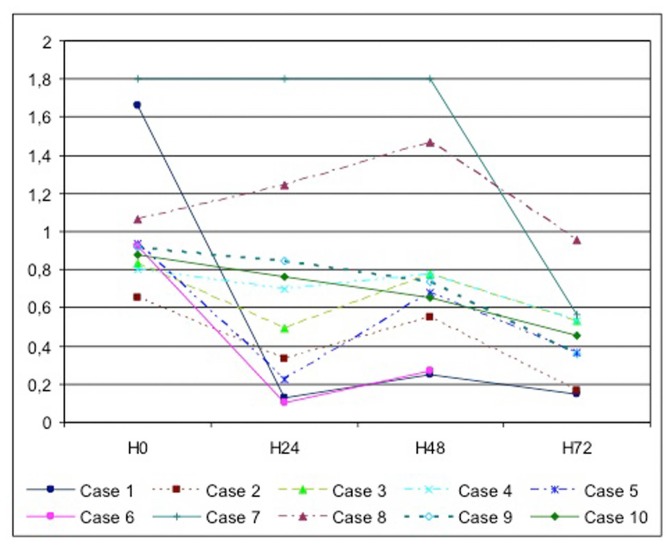
Viability of human pancreas tissue slices cultured *ex-vivo* for up to 3 days by a MTT test at each step of the procedures at baseline, H24, H48 and H72. All data are given as the means of at least three slices.

Viability of the tissue was analyzed by a MTT test reflecting the mitochondrial activity. Even if MTT levels decreased during the culture process, they revealed a preserved cell activity. Under hyperoxic conditions of culture, the median MTT levels for all cases at each culture step were H0 (0.925 [0.656-1.8]), H24 (0.597 [0.131-1.8]), H48 (0.707 [0.253-1.8]), H72 (0.453 [0.15-0.956]). No significant difference was observed between all cases at each different step of the culture (H0, p=0.56; H24, p=0.73; H48, p=0.61; H72, p=0.82) (Figure 2).

**Figure 3:**
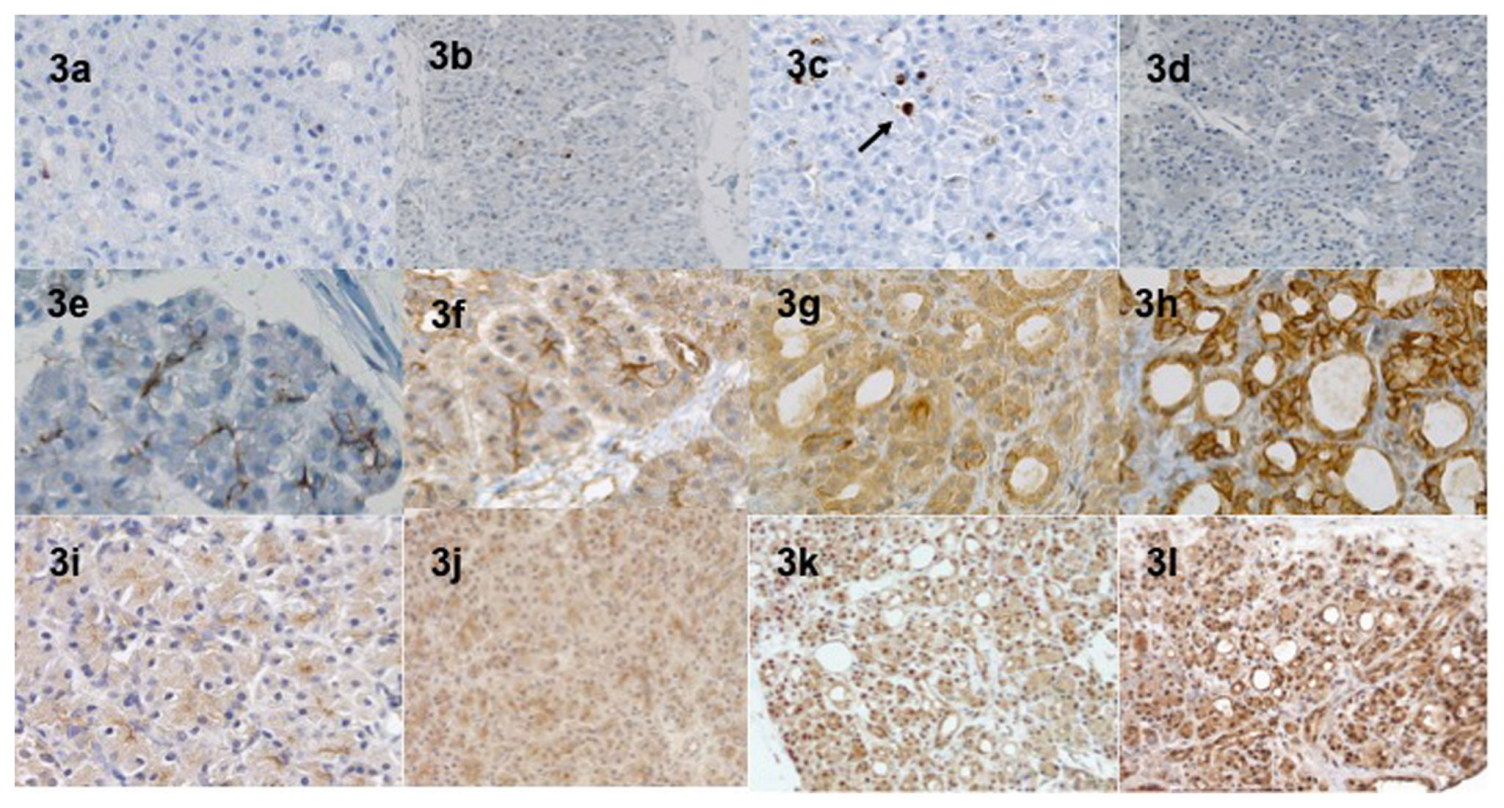
Immunohistochemical expression of caspase-3 antibody at baseline (a), H24 (3b), H48 (3c) and H72 (3d) in hyperoxic conditions. At H72 (3d), less than 5 cells per HPF expressed the caspase-3. Other types of cells (islets, ductal) did not express caspase-3 at any time. The membranous CA9 and the nuclear Hif-1α expression in acinar cells was studied at baseline (3e and 3i), H24 (3f and 3j), H48 (3g and 3k) and H72 (3h and 3l). The intensity greatly increased in dedifferentiated areas with a gradual appearance.

Apoptosis was assessed by the activated caspase-3 expression. The median number of positive acinar cells per HPF was not different at the different times of the culture process in normoxic and hyperoxic conditions (Table 1). At H72, less than 5 cells per HPF expressed the caspase-3 (Figure 3). Other types of cells (islets, ducts) did not express caspase-3 at any time.

**Table 1:** Characteristics of the caspase-3 immunochemical staining.

**Conditions of culture**	**H0 n=10**	**H24 n=10**	**H48 n=10**	**H72 n=10**	**p**
**90% O2 n=10**	0	1 [0-1]	2 [0-3]	5 [1-7]	NS
**21% O2 n=5**	0	0 [0-1]	1 [0-4]	3 [1-5]	NS

Mean total number of caspase-3 acinar cells per high-power field (HPF x 40 objective) counted in a minimum of 3 HPFs. The result corresponded to the median number of positive acinar cells per field in all cases.

NS, none significant

**Figure 4:**
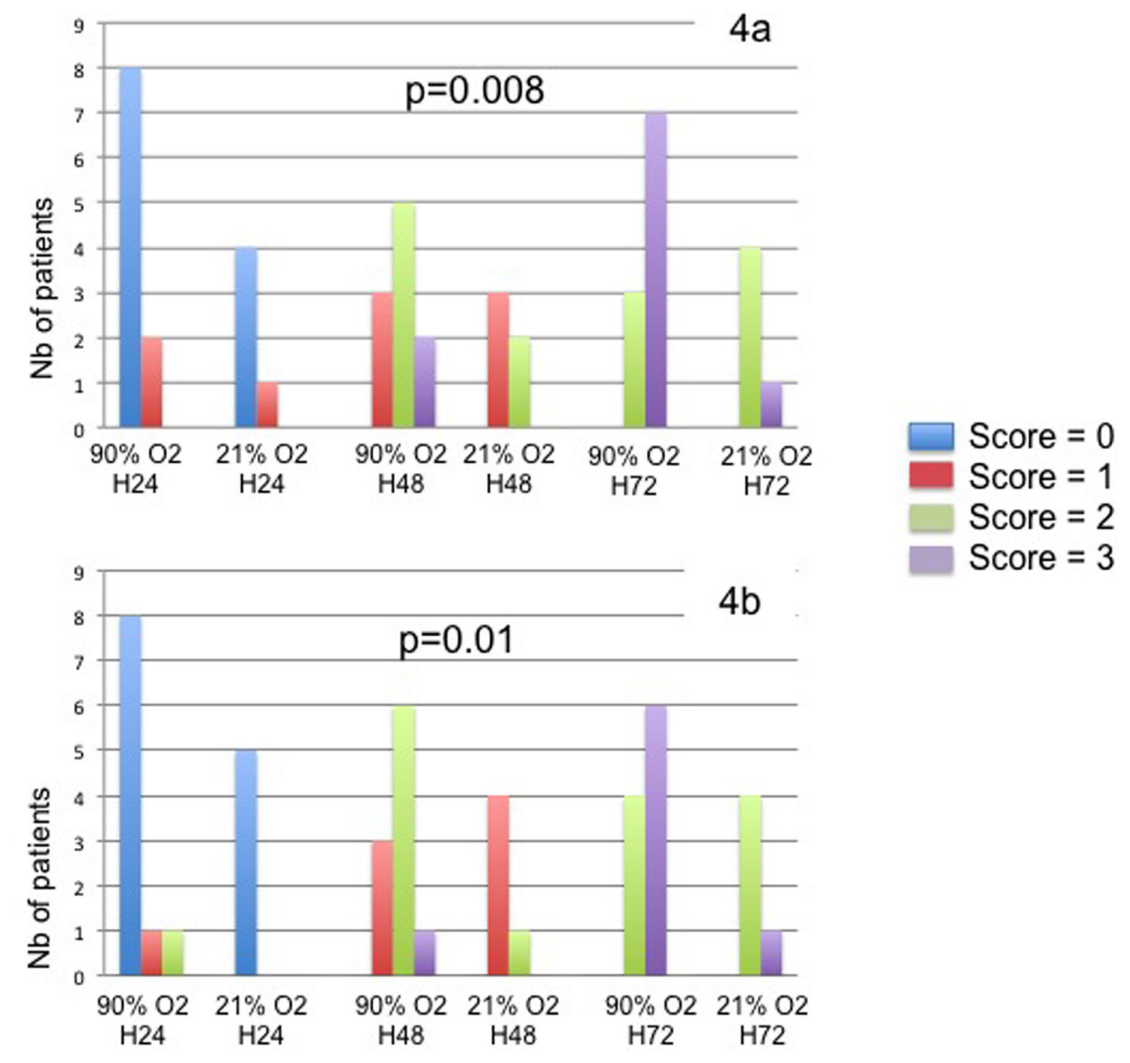
Expression of the CA9 (4a) and the HIF-1α (4b) in acinar cells under hyperoxic and normoxic conditions of culture. The intensity of staining was evaluated as negative scored as 0, weak scored as 1, moderate scored at 2 and strong scored as 3. Results are expressed as the number of cases for each category of the score.

**Figure 5:**
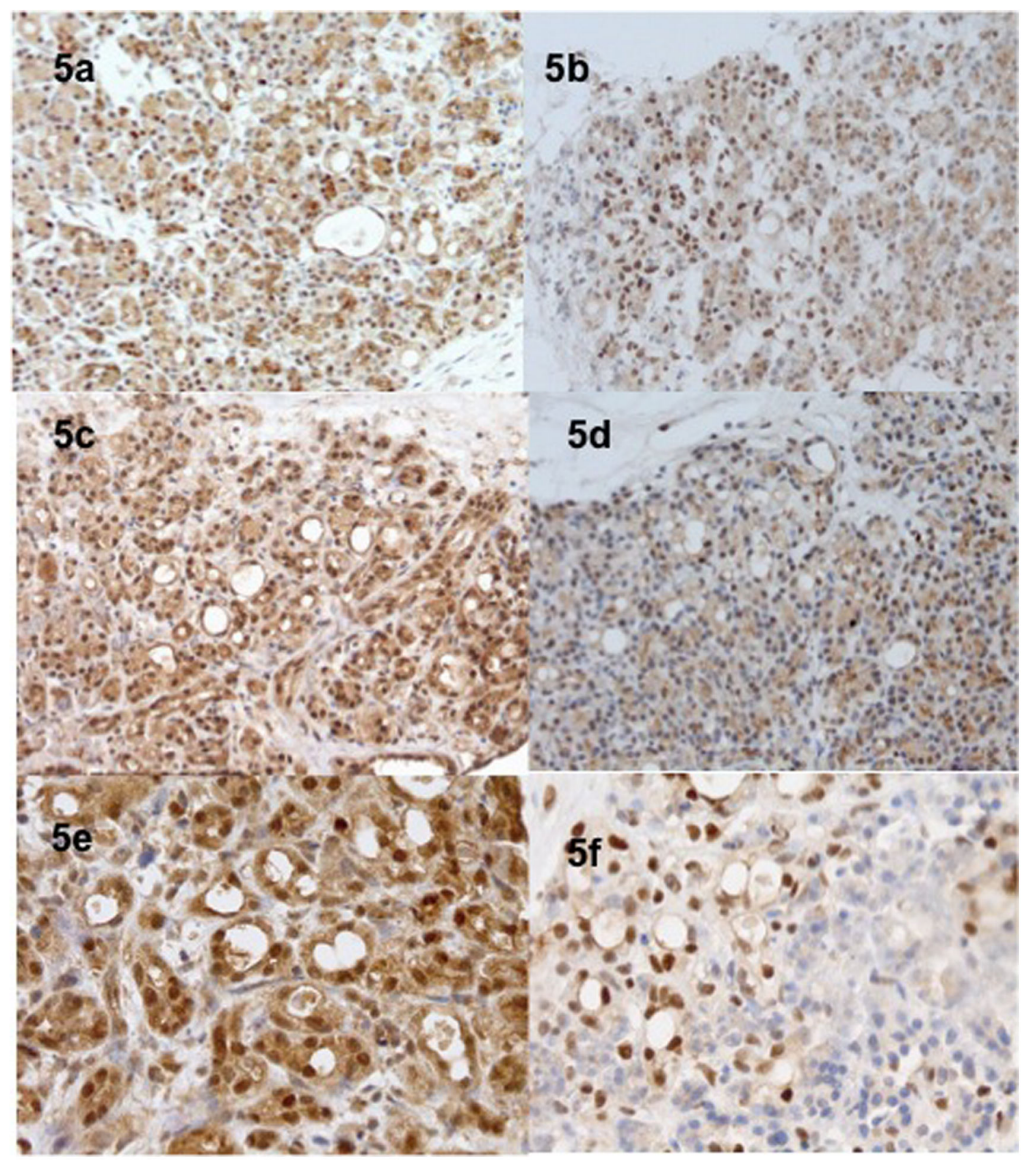
Expression of the Hif-1α in hyperoxic conditions (H24, 5a; H48, 5c and H72, 5e) and in normoxic conditions (H24, 5b; H48, 5d and H72, 5f). Hyperoxia conditions promoted a more intense expression of the hypoxia pathways.

The activation of the hypoxia pathways was evaluated by the membranous CA9 and the nuclear HIF1-α expression in acinar cells. At baseline, the intensity of the CA9 and the Hif- α expression was nil in all cases (hyperoxic and normoxic conditions). The results of the semi quantitative score are presented in the Figure 4. The expression of these two markers was correlated and time dependant during the culture process. The intensity of expression greatly increased at the periphery of necrotic areas and in dedifferentiated areas with a gradual appearance. Under hyperoxic conditions, a high expression was specifically observed at the periphery of the slices (Figures 3 and 5).

### Tissue differentiation

**Figure 6:**
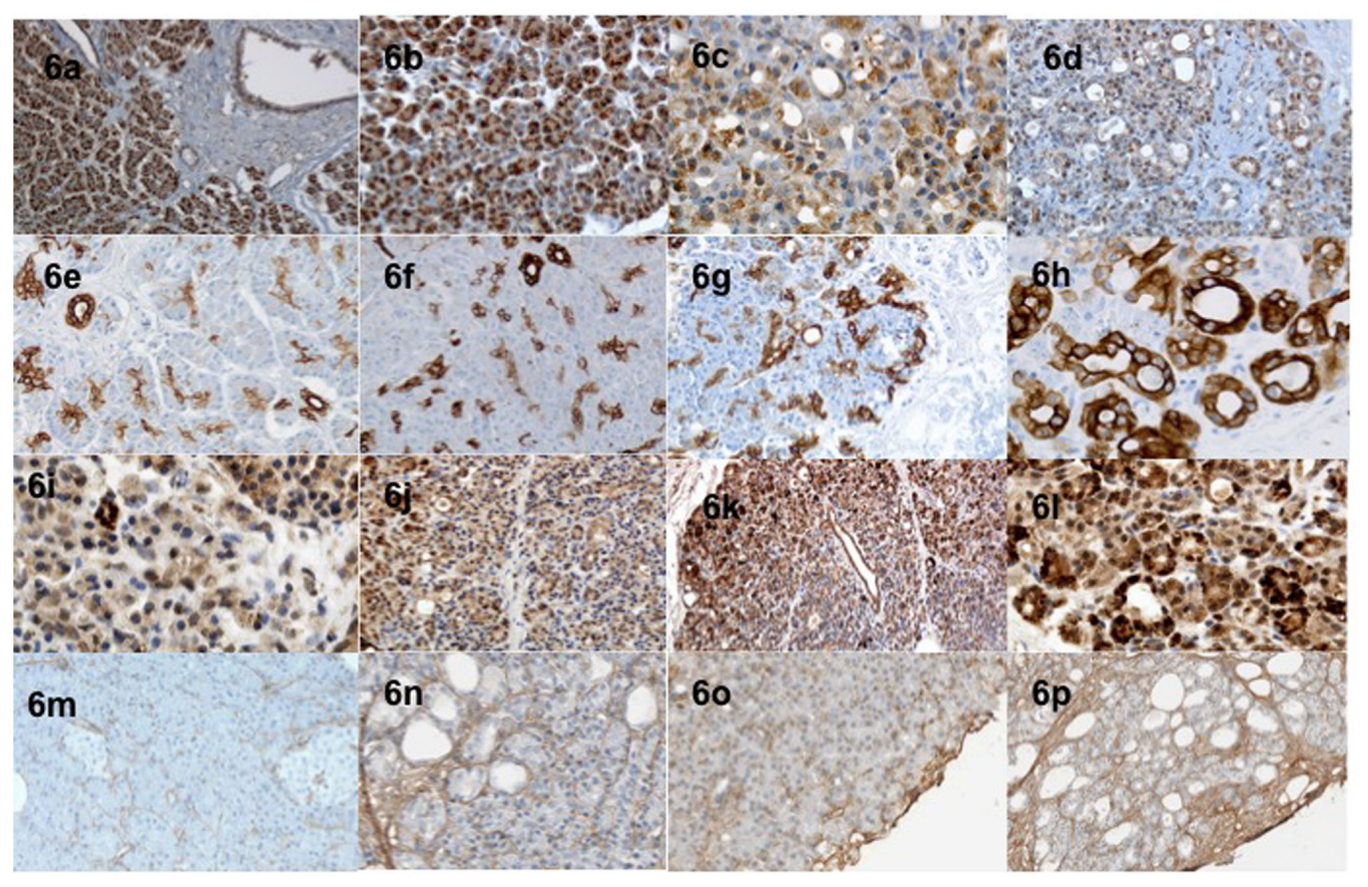
Trypsin expression intensity in acinar cells gradually decreased in the dedifferentiation area (baseline (6a), H24 (6b), H48 (6c) and H72 (6d)). A contrario, an increased expression of ductal and acinar expression of CK7 was progressively observed during time of culture (Baseline (6e), H24 (6f), H48 (6g) anf H72 (6h)). The TGF-β and the collagen-I expression in pancreatic tissue at baseline (6i and 6m) increased during the culture time (H24, 6j and 6n) especially at peripheral of the slice (H48, 6k and 6o and H72, 6l and 6p).

**Figure 7:**
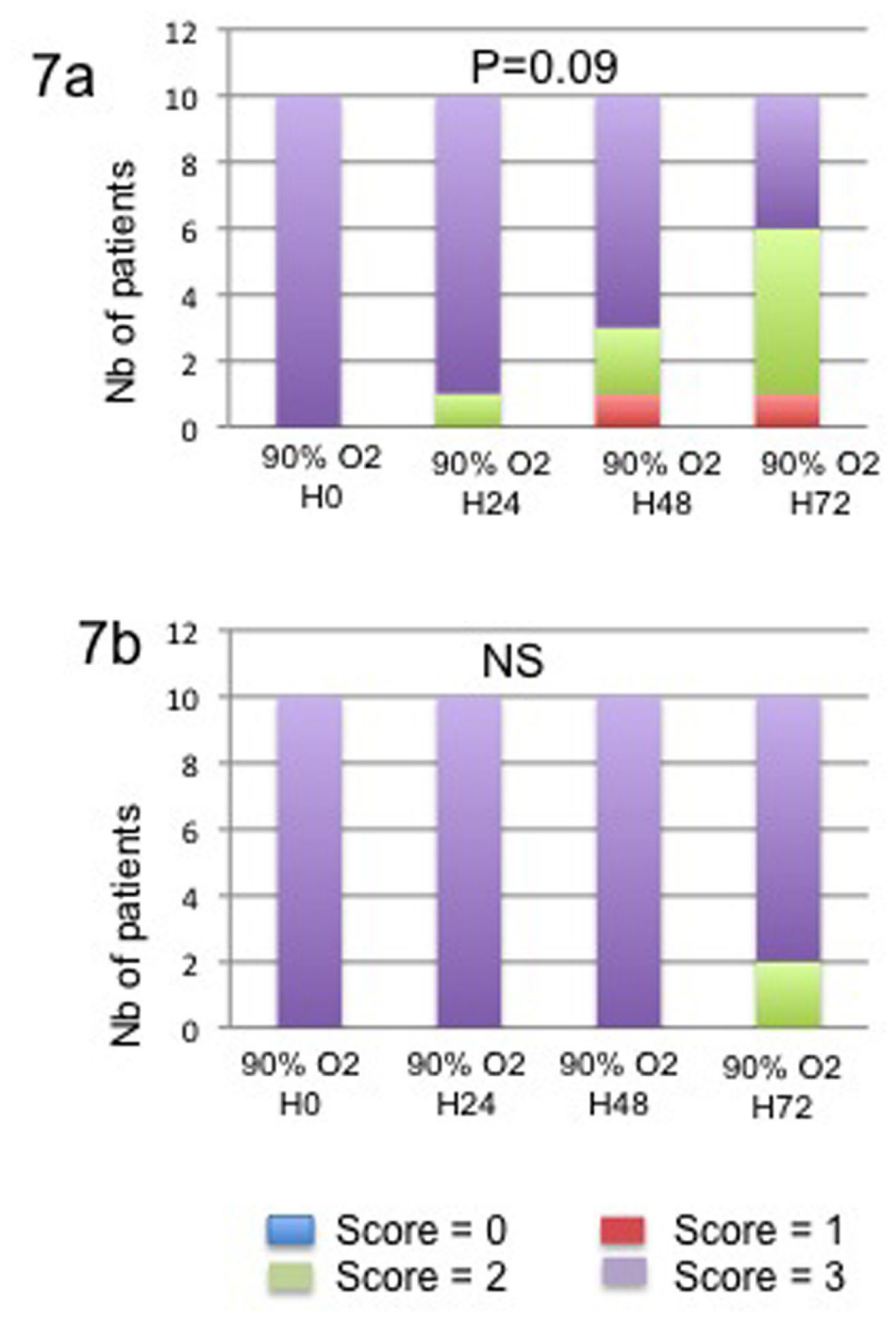
Expression of trypsin (7a) in acinar cells and CK7 in ductal cells (7b) under hyperoxic conditions of culture. The intensity of staining was evaluated as negative scored as 0, weak scored as 1, moderate scored at 2 and strong scored as 3. Results are expressed as the number of cases for each category of the score.

Cell differentiation was analyzed in the different types of cells composing the pancreatic tissue. In hyperoxic and normoxic conditions, endocrine islet cells maintained their normal insulin phenotype without any difference in intensity of expression during culture. A strong immunostaining for insulin (score 3) was observed in all cases at H0, H24, H48 and H72 (NS). In exocrine tissue, the number of cases with a strong expression of trypsin (score 3) in acinar cells was 10, 9, 7 and 4 at H0, H24, H48 and H72, respectively (p=0.09). Normal ducts were consistently positive for CK7 and CA9 staining. The decrease in intensity was gradual in the dedifferentiation area with loss of trypsin expression. A contrario, an increase expression of acinar expression of CK7 and CA9 was progressively observed in the same areas, underlying in parallel the switch for a ductal phenotype (Figures 3 and 6). The results are summarized in the Figure 7.

### PSC proliferation and activation

**Figure 8:**
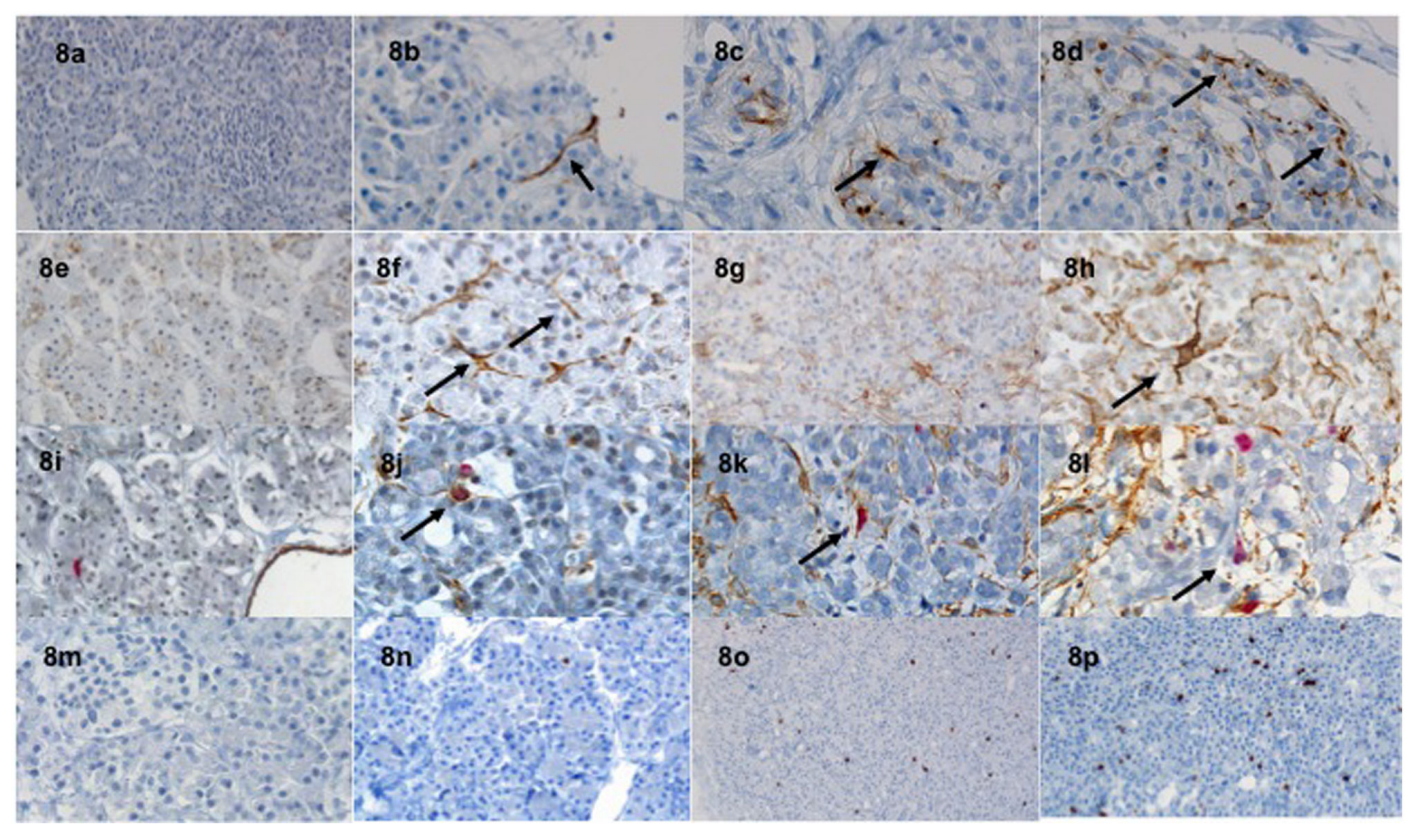
Nestin expression of PSC (black arrows) at baseline (8a), H24 (8b), H48 (8c) and H72 (8d). Smooth muscle actin expression of PSC (black arrows) at baseline (8e), H24 (8f), H48 (8g) and H72 (8h). Double Ki67/α-SMA expression of PSC was performed. The nuclear Ki67 and the cytoplasmic α-SMA expression (black arrows) appeared as red and brown immunostaining, respectively (baseline, 8i; H24, 8j; H48, 8 k and H72, 8l). The Ki67 nuclear staining of PSC was observed (baseline, 8m; H24, 8o; H48, 8p and H72, 8q).

**Figure 9:**
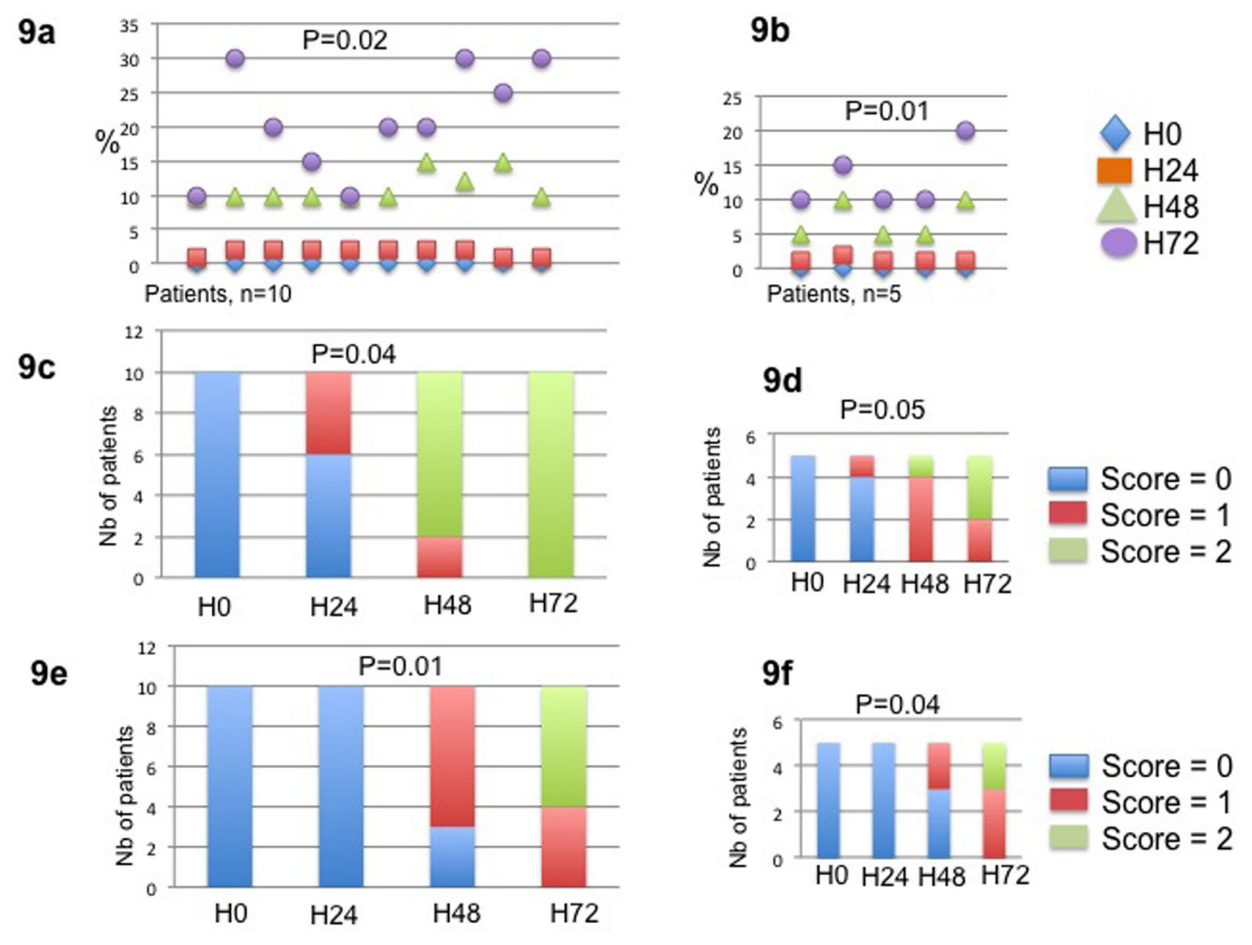
Immunochemical expression of Ki67, Nestin and Actin smooth muscle under 90% O2 (9a, 9c and 9e) and 21% O2 (9b, 9d and 9f) conditions of culture. The Ki67 expression was assessed by counting the mean number of Ki67 PSC per high-power field (HPF x 40 objective) in a minimum of 3 HPFs. The result corresponded to the median number of PSC per field in all cases. The nestin and α-SMA expression was evaluated using a semi quantitative score. The score corresponded to 0: no positive PSC staining per HPF; 1: rare positive PSC per HPF and 2: several positive PSC per HPF. The number of positive PSC represented the mean of PSC counted in a minimum of 3 HPFs. Results are expressed as the number of cases for each category of the score.

PSC proliferation status was evaluated by Ki67 expression. At baseline, PSCs did not express Ki67. In hyperoxic conditions, the median number of Ki67 positive PSC per HPF was 2 [1,2], 10 [10-15] and 20 [10-30] at H24, H48 and H72, respectively (p=0.02). The Ki67 expression by PSC was confirmed by a double Ki67/α-SMA immunostaining (Figure 8). Ki67 staining was correlated to the α-SMA expression and was higher around ducto-acinar dedifferentiation morphological changes (Figure 8). The activation of PSC was confirmed by the increased expression of α-SMA during the culture process. The median semi quantitative score of α-SMA expression was nil in all patients at baseline. In hyperoxic conditions, the score was 0, 1 and 2 in 6, 4, 0 cases at H24, 0, 2, 8 at H48 and 0, 0, 10 at H72, respectively (p=0.04). The α-SMA expression of PSC focused in areas surrounding ducto-acinar dedifferentiation and in the periphery of slices. A contrario, in normoxic conditions, the expression of the α-SMA was homogenous and more intense around ducto acinar metaplasia. Nestin and α-SMA expressions were positively correlated (Figure 8). The number of PSC stained with nestin was higher at H48 and H72 (score 1 and 2 in 7, 0 and 4, 6 of the cases, respectively). The score was negative at baseline and at H24. The GFAP expression was negative in all cases and at each step of culture. The TFG-β secretion by the acinar cells gradually increased during time of culture, especially around acinoductal metaplasia and at peripheral of tissue slice in hyperoxic conditions (Figure 6). At basal state, TFG- β was only faintly detected in islets cells. The collagen-I expression was evaluated in each step of culture time. Three areas were analysed at each step for each slice. The percentage of positive tissue area significantly increased between each step of culture for each specimen. The mean percentage of positive tissue area was 2.41%, 10.7%, 21.59% and 33% at H0, H24, H48 and H72, p<0.001. All the results are summarized in the Figure 9.

## Discussion

This study demonstrates that human pancreatic slices can be cultured for three days without obvious signs of necrosis or apoptosis and with preserved physiological properties. Morphological analysis showed gradual appearance of acinoductal metaplasia, correlated to time culture and cell stress. The acquisition of the ductal phenotype was confirmed by CK7 and CA9 *de novo* expression. Apoptosis was limited, and only rare acinar cells expressed caspase-3 at H48 and H72 (NS). Analysis of PSC proliferation using Ki67 index underlined significant proliferation of PSC (p=0.02) from H48, which was paralleled with PSC transdifferentiation to a myofibroblastic phenotype as demonstrated by *de novo* expression of smooth muscle actin, especially around HIF1-α positive areas.

Precision-cut slice techniques were developed two decades ago for metabolic and toxicological studies in various cell types (liver, kidney, hippocampal...) in order to analyse metabolism and physiopathological mechanisms in normal organ tissue[15-17]. The main interest of this technique is that slices contain all cell types of the tissue in their natural environment, with intercellular and cell-matrix interactions remaining intact to be more representative of the *in vivo* situation. Using an automatic tissue slicer, no tissue damages were observed at baseline and the tissue architecture was preserved. This method allowed comparable slices in size and viability, with minimal loss of tissue. The tissue was preserved in its fresh, healthy and native cellular milieu, unperturbed by the damaging mechanical and enzymatic stresses inherent to conventional pancreatic cell isolation or dispersion procedures. Moreover, slices were homogenous and reproducible between specimens. Only one study reported pancreatic precision slices techniques for examining virus-host interactions in pancreatic normal and adenocarcinoma tissue. Pancreatic normal and cancer tissue slices were cultured for up to 6 days, while retaining viability and moderate to good morphological features[18].

Allowing the maintenance of normal acinar architecture and cell–cell interactions within their original matrix, this model has recently been applied to the study of stellate cell activation in the liver[13,19]. It appeared especially well suited to investigate the remodelling of tissues and the activation/deactivation of the liver stellate cells. Culture of *ex vivo* pancreatic explants could be the response to the lack of normal human pancreas cells, and the difficulties to find a representative model of the *in vivo* situation. Indeed all published studies underlined the variations between murine and human PSC species as well as donor-dependent variances. The development of immortalised PSC proposed, as an alternative model to study pancreatic stroma, could be useful for molecular signalling or for models that require cells to remain viable for a longer period of time. But differences between immortalised and primary PSC did not allow to confirm the results obtained in clinical situation[5,12]. Moreover, the main characteristic of the PSCs is the pivotal role in fibrogenesis and tumor growth. It underlines the permanent interactions between PSCs and other cell types in the pancreas. This model goals to maintain as far as possible these biochemical interactions and allows us to study the PSC reactions to cellular stress situation.

This study confirmed that pancreatic culture slices are feasible with reproducible conditions, few variations between cases (no significant differences of the MTT test at each step of the culture) and optimized tissue viability at three days. The evaluation of apoptosis by caspase-3 immunostaining did not show significant differences during the culture process and less than 5% of the cells were positive at H72. In normoxic conditions, morphological changes were homogenous and less frequent. In hyperoxic conditions, at microscopic examination, the inner cell layers were more viable than peripheral ones, confirming that oxygen and nutrient diffusion through the slices does not seem limiting. The HIF1-α immunostaining increased during culture at each step, was located in small areas of necrosis and was correlated to the morphological changes as acinoductal metaplasia. These observations confirmed that hyperoxia promoted the activation of the hypoxia pathways (especially at peripheral) as a response to cellular and oxidative stress.

In major pancreatic diseases such as pancreatitis and adenocarcinoma, a process called acino-ductal metaplasia was described as the replacement of acinar cells by duct cells. Two different contributing mechanisms have been recognized, i.e elimination of acinar cells by apoptosis in combination with selective expansion of duct cells or the transdifferentiation of acinar to ductal cells. Based on genetic models and in vitro culture systems, murine pancreatic acinar cells have been attributed the capacity to transdifferentiate into duct cells[20]. However the inherent plasticity of rodent acinar cells and their capacity to get dedifferentiated in experimental pancreatic cancer model could question about the same properties of human acinar cells in *in vivo* situation[21]. A few studies were published using cultured human exocrine tissue obtained from surgical specimen. They reported that acinar features (expression of digestive enzymes) were rapidly lost, while ductal features (cytokeratin 19 expression) concomitantly increased. However, none of these studies has used cell-tracing method and their conclusions are based on indirect evidence[22-25]. A recent study reported a genetic lineage tracing methods to study the fate of human acinar cells in culture. Authors confirmed that human acinar cells can transdifferentiate into cells that express specific ductal markers, such as cytokeratin 19. Within 1 week of culture, all surviving acinar cells had acquired a ductal phenotype[22]. In this study, the acinoductal metaplasia was associated to a dedifferentiation phenomenon with a gradual appearance of ductal phenotype at each step of culture. In the specific model of precision slice culture, the dedifferentiation phenomenon was mainly observed around necrosis or peripheral proximity and was correlated to the gradient of hypoxia pathways activation by increasing CK7 and decreasing trypsin immunostaining. These findings underlined the need of pancreatic injury to promote these changes and the role of cellular stress due to hyperoxic conditions. Furthermore, our study shows a close topographical correlation between acinoductal metaplasia and activation of PSC. Whether these 2 phenomenon are self-stimulated or derive from a common stimuli remains to be investigated. Chronic pancreatitis is a well known risk factor of pancreatic cancer, the role of recurrent early dedifferentiation changes could be hypothesized as one of the physiopathological processes involved in the development of precancerous lesions[26].

Areas with intense activation of hypoxia pathways were also the preferential location of PSC activation confirmed by a higher α-SMA and nestin expression. PSC proliferation was demonstrated by a significant increase of Ki67 expression during the culture process. The role of PSC in fibrogenesis and oncogenesis is now well-known, especially in the fibrogenesis/fibrolysis balance. During pancreatic injury, PSC are transformed (in response to factors as oxidant stress, cytokines, growth factors and toxins such as alcohol) from their quiescent state to an activated myofibroblast-like phenotype that synthesises and secretes excessive amounts of ECM proteins[4,9]. In *in vitro* studies, the role of hypoxia pathways in PSC activation was analyzed. Activity of PSCs’ increased significantly and the secretion of periostin, collagen-I, fibronectin, and vascular endothelial growth factor (VEGF) doubled. PSCs were the dominant producers of VEGF and increased endothelial cell growth in the peritumoral stroma. Moreover they contributed to the fibrotic/hypoxic milieu through abnormal extracellular matrix deposition and by amplifying endostatin production of cancer cells. Acute pancreatitis is mainly characterized by hypoxic states and oxidative stress due to inflammation changes. The present study underlined the very early activation of PSC in case of hypoxic pathways activation and might account for chronic PSC activation during the states of minimal chronic inflammation in hypoxic conditions.

In conclusion, thick slices *in vitro* culture of normal human pancreas is possible with optimized cell viability at 72 hours. Activation of PSC occurs very early in close relationship with cellular stress and in association with acinoductular metaplasia. Our model suggests that cell stress (as in inflammation state) may participate in the early stages of fibrogenesis and oncogenesis by activating PSC. Organotypic culture represents an important opportunity and a useful model allowing studying the physiopathological mechanisms involved in PSC activation, and the effects of various antifibrotic drugs and therapeutic targets to inhibit/revert PSC activity.
